# Effect of mouth rinses on the color stability of IPS EMAX, Zirconia and PEEK CAD materials: An *in vitro* study

**DOI:** 10.6026/973206300213336

**Published:** 2025-09-30

**Authors:** Kritika Kar, Alka Gupta, Harsh Chansoria, Mukesh Soni, Arti Shyam, Shivani Gupta

**Affiliations:** 1 Department of Prosthodontics and Crown and Bridge, Government College of Dentistry, Indore, Madhya Pradesh, India; 2 Department of Orthodontics and Dentofacial Orthopaedics, Sri Dharmasthala Manjunatheshwara College of Dental Sciences, Dharwad, Karnataka, India

**Keywords:** CAD-CAM, chlorhexidine, color stability, IPS e.max, zirconia, PEEK

## Abstract

The color stability of three restorative materials (IPS e.max, Zirconia and PEEK) after exposure to various mouthrinses is of interest. Samples were immersed in Listerine, Chlorhexidine, Bioayurveda herbal mouthwash and distilled water for 180 hours, equivalent to 15 years of exposure. Results indicated that IPS e.max exhibited the highest color stability, while Zirconia and Polyether Ether ketone (PEEK) showed more significant color changes. Listerine and Chlorhexidine had the most pronounced effects on the materials' color, while herbal mouthwash had a lesser impact. Thus, we show the importance of selecting suitable materials and mouthwashes in ensuring long-lasting aesthetic restorations.

## Background:

Recent advancements in dental materials have led to a growing demand for restorations that provide both optimal functionality and superior aesthetic results. As patient expectations regarding appearance have increased, the field of aesthetic restorations has evolved rapidly, emphasizing both visual appeal and practicality [1]. Modern dentistry demands restorations that not only look natural but also remain durable over time [2]. A key challenge is ensuring that dental materials match the natural color of teeth and maintain long-term durability [3]. Factors such as plaque buildup, stains from food and beverages, uneven surfaces and material degradation contribute to the fading of restorative color. Consumption of tea, coffee and mouthwash, as well as exposure to extreme temperatures, can accelerate these changes [4]. Despite improvements, creating restorations that maintain both beauty and longevity remains a challenge. Polymers, like tooth-colored composites, have been prone to fading since their introduction in the 1940s, especially in anterior restorations where appearance is crucial [5]. Porcelain has been used for over a century, but its main drawback is its inability to withstand the stresses of the oral environment. Metal-ceramic restorations, which combine the beauty of ceramics with the strength of metal, have solved many issues but introduce others, such as allergic reactions and gum discoloration [6]. This has driven the development of all-ceramic restorations, including lithium disilicate and zirconia, which offer both durability and aesthetics, addressing the issues of metal-ceramics [7]. Advanced techniques, such as CAD- CAM machining and hot pressing, further enhance the fit and precision of these restorations, thereby reducing the risk of cement cracks and gaps [8].

Zirconia, especially yttria-stabilized zirconia (YSZ), is known for its strength and safety, withstanding heavy loads and bending forces. However, due to its opacity achieving a perfect color match for zirconia remains a challenge, which affects patient satisfaction and treatment outcomes [9]. Lithium disilicate ceramics, such as IPS E.max, are popular for anterior restorations due to their ability to vary in color and translucency. However, these materials can break down under certain conditions, such as posterior teeth restoration; also exposure to mouthwash, leading to discoloration [10]. PEEK (polyetheretherketone) is another promising material for dental restorations due to its biocompatibility, chemical stability and ability to endure extreme temperatures. When combined with ceramic fillers, PEEK can be used to create durable, aesthetically pleasing prosthetics [11]. Color stability remains a significant concern in restorative dentistry, as factors such as chemical reactions and exposure to staining substances, including nicotine and coffee, can affect the material's appearance [12]. Advancements in technologies, such as spectrophotometers and standardized color schemes, have improved the ability to assess color stability, providing more accurate and objective results for both patients and dentists [13, 14]. Despite ongoing advancements, color stability and material durability remain key areas of focus in dental restoration, requiring continued research and development. Therefore, it is of interest to investigate the impact of various mouthrinses on the color stability of IPS e.max CAD, Zirconia CAD and PEEK CAD blocks.

## Methodology:

The methodology of this study involves several critical steps, including sample design, sample fabrication, immersion of samples and spectrophotometric analysis. Initially, rectangular cross-section samples of 14mm x 12mm x 2mm were designed using NX (UNIGRAPHICS) software for IPS e.max, Zirconia and PEEK materials and a standard tessellation language (STL) file was created for fabrication. Forty samples of each material were then fabricated using a CAD/CAM system and randomly allocated to twelve different groups. For IPS e.max, the samples were sintered at 850°C for 30 minutes according to manufacturer's instructions. For Zirconia, the pre-sintered samples underwent a 14-hour sintering cycle at 1530°C. For PEEK, samples were polished using silicon carbide abrasive papers. After fabrication, the samples were immersed in one of four solutions: distilled water (control), Listerine, Chlorhexidine 0.2%, or Bioayurveda herbal mouthwash-to simulate 15 years of use till 180 hours, since exposure of 12 hours has been reported to be equivalent to 1 year of 2 min daily mouthrinse use. The solutions were being replenished every 12 hours. Following immersion, the samples were washed, dried and analyzed using a VITA Easyshade spectrophotometer to measure reflectance, recording values based on the CIELAB color system. Color shift values (ΔE) were calculated to assess the impact of mouthrinses on the color stability of the materials. This methodology enables a comprehensive evaluation of how various mouthrinses impact the long-term color stability of dental restorative materials.

## Results:

The comparison of ΔE values between IPS e.max, Zirconia, and PEEK for each group is presented in the following figures and tables. In the control group (Figure 1 - see PDF), the ΔE values were highest for Group 9 (PEEK), followed by Group 1 (IPS e.max) and then Group 5 (Zirconia). Group 5 demonstrated the least color change, although no statistically significant differences were found between the ΔE values of Groups 1, 5, and 9 (p-value > 0.05), as shown in Table 1 (see PDF). In the Listerine group (Figure 2 - see PDF), Group 10 (PEEK) exhibited the highest ΔE values, followed by Group 6 (Zirconia) and then Group 2 (IPS e.max). Significant differences were observed between Group 2 and both Groups 6 and 10 (p-value < 0.05), with Group 2 showing significantly lower ΔE values than the other two groups. Post hoc analysis revealed that Group 6 also showed significantly lower ΔE values compared to Group 10 (p-value < 0.05), as detailed in Table 2 (see PDF). For the Chlorhex group (Figure 3 - see PDF), Group 11 (PEEK) had the highest ΔE values, followed by Group 7 (Zirconia) and Group 3 (IPS e.max). Significant differences were observed between Group 3 and both Groups 7 and 11 (p-value < 0.05), with Group 3 showing significantly lower ΔE values. Group 7 also had considerably lower ΔE values compared to Group 11, as presented in Table 3 (see PDF). In the Herbal group (Figure 4 - see PDF), Group 12 (PEEK) exhibited the highest ΔE values, followed by Group 8 (Zirconia) and Group 4 (IPS e.max). Group 4 demonstrated the least color change, with significantly lower ΔE values than Groups 8 and 12 (p-value < 0.05), as shown in Table 4 (see PDF).

The ΔE values for IPS e.max (Figure 5 - see PDF) were highest in Group 2 (Listerine), followed by Group 3 (Chlorhex), Group 4 (Herbal), and Group 1 (Control). Significant differences were found between Group 2 and the other groups, with post hoc analysis revealing significant differences between Group 1 and Group 2, Group 1 and Group 3, and Group 2 and Group 4, as summarized in Table 5 (see PDF). For Zirconia (Figure 6 - see PDF), the ΔE values were highest in Group 7 (Chlorhex), followed by Group 6 (Listerine), Group 8 (Herbal), and Group 5 (Control). Significant differences were observed between Group 7 and all other groups (p-value < 0.05), with Group 7 showing the greatest color change, as shown in Table 6 (see PDF). In the PEEK group (Figure 7 - see PDF), Group 11 (Chlorhex) had the highest ΔE values, followed by Group 10 (Listerine), Group 12 (Herbal), and Group 9 (Control). Significant differences were noted between Group 9 and all other groups (p-value < 0.05), with Group 9 showing the least color change, as presented in Table 7 (see PDF). Finally, the ΔE values were converted to National Bureau of Standards (NBS) units for clinical interpretation. The NBS unit conversion revealed that for most materials, the color change was classified as "slight change" or "perceivable," with the largest color shifts occurring in the Listerine and Chlorhex groups. The corresponding NBS units and clinical interpretations are provided in Table 8 (see PDF) and Table 9 (see PDF).

## Discussion:

The loss of tooth structure, particularly the absence of anterior teeth, has long been recognized as a source of physical, functional and even social challenges, leading to the demand for durable and aesthetically pleasing restorative materials. Today, the value of natural-looking dental restorations is widely acknowledged, with many patients seeking metal-free alternatives [15]. Modern dentistry, both in terms of restoration and aesthetics, has risen to meet these needs, facilitated by advancements in CAD-CAM technology. This technology has allowed for the fabrication of restorations that offer enhanced mechanical properties and aesthetic outcomes. CAD-CAM systems have made metal-free monolithic restorative materials increasingly popular due to their natural appearance and the high mechanical strength they provide even at low material thicknesses [16]. Among the various materials, monolithic zirconia stands out for its excellent mechanical strength, reasonable aesthetic qualities and reduced production time compared to veneered restorations. However, despite these advantages, achieving a precise shade match with monolithic zirconia remains a significant challenge [17]. Lithium disilicate (LD) is a widely used glass-ceramic due to its outstanding optical properties, high strength and ease of fabrication. Additional benefits of lithium disilicate include improved marginal integrity, lower porosity and the ability to form net shapes through pressing. While LD is considered a versatile material for indirect restorations, caution should be taken when using it for bruxism cases with high occlusal stress [18]. PEEK is the main ingredient in Bio HPP, a high-performance polymer. It has amazing physical properties, low specific weight, low plaque affinity and high biocompatibility. People have used it for various dental restorations, such as temporary abutments, rpd frameworks and dental implants. It also addresses many of the challenges associated with metal-resin restorations, making it a wonderful alternative for restoring the full arch. PEEK is a great material, but it lacks an aesthetically pleasing appearance; therefore, it needs to be veneered, especially in the aesthetic zone [19].

Several factors can influence the color of restorations in the mouth, including temperature, humidity, diet, smoking and the use of mouthwash. Researchers have demonstrated that acidic beverages, such as coffee, cola and orange juice, as well as stomach acid, can alter the appearance and texture of tooth-colored restorative materials. Restorations for teeth must not only be esthetically pleasing but also function well to bear occlusal loads [20]. Most commonly used mouthrinses is chlorhexidine to improve oral health, but it can also cause their teeth and dental prosthesis to stain. Essential oil mouthwashes, such as Listerine, can also help prevent plaque and gingivitis from worsening. However, they are acidic and contain alcohol, which can harm all ceramic restorations, resulting in porosity and staining [21]. Herbal mouthwashes are an excellent choice because they contain plant extracts that are known to kill bacteria and reduce inflammation. These products often contain bioactive substances, such as catechins and tannins that can help maintain healthy gums. They can also help by fighting free radicals and inflammation. Till date no research concerning the effect of these herbal mouthrinses on the colour stability of dental restorations has been documented, so further investigation is necessary. Concerns persist regarding the color stability of dental materials have led researchers to examine the effects of various beverages on ceramics, polymeric resins, composites and other materials. However, there is limited research on the influence of mouthwashes on CAD-CAM materials such as IPS e.max, monolithic zirconia and PEEK [22, 23]. The aim of this study was to evaluate the color stability of three CAD-CAM restorative materials-IPS e.max, zirconia and PEEK-after exposure to different mouthwashes. Samples were designed using CAD software, then sintered and finished according to the manufacturer's instructions. The samples in four different solutions were placed for 180 hours, simulating 15 years of exposure, to test how well the materials retained their colour integrity. The control was distilled water and the other solutions were Listerine, Chlorhexidine 0.2% and Bioayurveda herbal mouthwash. In colour science, an object's colour is measured by assigning specific numerical values to it. In the CIELab colour model, L* represents lightness, a* measures the red-green balance and b* indicates the yellow-blue balance. A spectrophotometer (Vita Easyshade) utilizing CIE Lab coordinate system was used to determine how the colors of the samples changed when they were immersed in different mouthrinse solutions. The ΔE formula was used to determine the change in color.

ΔE = [(ΔL)^2^ + (Δa)^2^ + (Δb)^2^] 1/2

Within the limitations of this *in vitro* study, it can be concluded that exposure to different mouthrinses has a significant impact on the colour stability of CAD-CAM restorative materials. IPS e.max demonstrated the greatest colour stability, followed by Zirconia and PEEK. Distilled water had the least impact on colour change, whereas Chlorhexidine and Listerine mouthrinses induced the most significant colour changes across all materials tested. Herbal mouthrinse (Bioayurveda) showed relatively less impact compared to Chlorhexidine and Listerine. These results suggest that material selection and patients' use of specific mouthrinses should be considered carefully, especially in aesthetically critical areas.

## Conclusion:

The composition of restorative materials and the chemical properties of mouthwashes significantly influence the long-term color stability of dental restorations. IPS e.max demonstrated the best color stability, followed by Zirconia, while PEEK may require further modification for high aesthetic demands. Future research should focus on improving PEEK's color stability through material composition enhancements and long-term clinical studies.
